# Development and Evaluation of a Mobile App Designed to Increase HIV Testing and Pre-exposure Prophylaxis Use Among Young Men Who Have Sex With Men in the United States: Open Pilot Trial

**DOI:** 10.2196/25107

**Published:** 2021-03-24

**Authors:** Katie B Biello, Jonathan Hill-Rorie, Pablo K Valente, Donna Futterman, Patrick S Sullivan, Lisa Hightow-Weidman, Kathryn Muessig, Julian Dormitzer, Matthew J Mimiaga, Kenneth H Mayer

**Affiliations:** 1 Department of Behavioral and Social Sciences Brown University School of Public Health Providence, RI United States; 2 Department of Epidemiology Brown University School of Public Health Providence, RI United States; 3 Center for Health Promotion and Health Equity Brown University School of Public Health Providence, RI United States; 4 The Fenway Institute Fenway Health Boston, MA United States; 5 Adolescent AIDS Program Children's Hospital at Montefiore Medical Center The Bronx, NY United States; 6 Department of Epidemiology Rollins School of Global Public Health Emory University Atlanta, GA United States; 7 Division of Infectious Diseases UNC School of Medicine University of North Carolina Chapel Hill, NC United States; 8 Department of Health Behavior Gillings School of Public Health University of North Carolina Chapel Hill, NC United States; 9 Department of Epidemiology Fielding School of Public Health University of California Los Angelas, CA United States; 10 Harvard Medical School Boston, MA United States; 11 Beth Israel Deaconess Medical Center Boston, MA United States

**Keywords:** HIV, men who have sex with men, pre-exposure prophylaxis, pilot study, mobile apps, mobile phone, mHealth

## Abstract

**Background:**

HIV disproportionately affects young men who have sex with men (YMSM) in the United States. Uptake of evidence-based prevention strategies, including routine HIV testing and use of pre-exposure prophylaxis (PrEP), is suboptimal in this population. Novel methods for reaching YMSM are required.

**Objective:**

The aim of this study is to describe the development and evaluate the feasibility and acceptability of the MyChoices app, a mobile app designed to increase HIV testing and PrEP use among YMSM in the United States.

**Methods:**

Informed by the social cognitive theory, the MyChoices app was developed using an iterative process to increase HIV testing and PrEP uptake among YMSM. In 2017, *beta* theater testing was conducted in two US cities to garner feedback (n=4 groups; n=28 YMSM). These findings were used to refine MyChoices, which was then tested for initial acceptability and usability in a technical pilot (N=11 YMSM). Baseline and 2-month postbaseline assessments and exit interviews were completed. Transcripts were coded using a deductive approach, and thematic analysis was used to synthesize data; app acceptability and use data were also reported.

**Results:**

The MyChoices app includes personalized recommendations for HIV testing frequency and PrEP use; information on types of HIV tests and PrEP; ability to search for nearby HIV testing and PrEP care sites; and ability to order free home HIV and sexually transmitted infection test kits, condoms, and lube. In theater testing, YMSM described that MyChoices appears useful and that they would recommend it to peers. Participants liked the *look and feel* of the app and believed that the ability to search for and be *pinged* when near an HIV testing site would be beneficial. Some suggested that portions of the app felt repetitive and preferred using casual language rather than formal or medicalized terms. Following theater testing, the MyChoices app was refined, and participants in the technical pilot used the app, on average, 8 (SD 5.0; range 2-18) times over 2 months, with an average duration of 28 (SD 38.9) minutes per session. At the 2-month follow-up, the mean System Usability Scale (0-100) score was 71 (ie, above average; SD 11.8). Over 80% (9/11) of the participants reported that MyChoices was useful and 91% (10/11) said that they would recommend it to a friend. In exit interviews, there was a high level of acceptability for the content, interface, and features.

**Conclusions:**

These data show the initial acceptability and user engagement of the MyChoices app. If future studies demonstrate efficacy in increasing HIV testing and PrEP uptake, the app is scalable to reach YMSM across the United States.

**Trial Registration:**

Clinicaltrials.gov NCT03179319; https://clinicaltrials.gov/ct2/show/NCT03179319

**International Registered Report Identifier (IRRID):**

RR2-10.2196/10694

## Introduction

### Background

HIV incidence remains high in the United States among young men who have sex with men (YMSM). In 2018, more than 20% of new HIV infections in the United States were among young people aged between 13 years and 24 years, with YMSM accounting for 83% of newly diagnosed HIV infections in this age group [1]. New HIV infections also disproportionately impact men who have sex with men (MSM) of color. In 2018, more than half (52%) of the new diagnoses of HIV among MSM aged between 13 years and 24 years were seen among Black individuals and 27% were identified among Latinx individuals [1]. In addition to experiencing a high HIV incidence, a higher proportion of YMSM living with HIV do not know that they are infected in comparison with their adult peers [2,3]. Moreover, individuals aged between 13 years and 24 years are less likely to be linked to HIV care upon diagnosis and present lower levels of viral suppression in comparison with older individuals [2,4,5]. Consequently, YMSM will have delays seeking effective treatment and are more likely to transmit HIV to others [6].

Overwhelming evidence shows that routine HIV testing and expanded use of pre-exposure prophylaxis (PrEP) would drastically reduce the population burden of HIV [7-13]; however, uptake of both interventions is suboptimal among young adults. For example, although the Centers for Disease Control and Prevention recommends that sexually active MSM be tested for HIV at least annually [14], data suggest that nearly half of YMSM reported not being tested for HIV in the past year and one-third reported they had never been tested [15]. In addition, PrEP awareness and uptake are low among younger people [16,17]. For example, only 5% of MSM aged between 18 years and 24 years with PrEP indications reported ever using PrEP, compared with 14% of those aged 25 years and more [18]. Furthermore, only 0.1% of PrEP prescriptions in the United States were given to individuals aged under 18 years [19]. Moreover, young individuals have lower levels of adherence to and retention in PrEP care after initial prescription [20-23], decreasing the impact of PrEP on HIV prevention among this group.

Risk taking, behavioral experimentation, and confronting a host of difficult choices regarding identity formation are all part of the normal developmental trajectory of adolescence and young adulthood [24]. In addition, beliefs about invincibility, sensation seeking, and the still-developing cognitive processes of adolescents may have a role in increased HIV risk-taking behaviors and a lower prioritization of prevention strategies for this age group [25-27]. Developing innovative ways to intervene to increase engagement in HIV prevention behaviors among youth is crucial, particularly interventions that are accessible and responsive to the diverse needs of youth.

Smartphones are used by nearly all youth in the United States, across race and social class, and as such are a direct way to *meet youth where they are* [28]. In addition, the use of mobile phone apps by YMSM is nearly ubiquitous; apps may offer unique opportunities for public health interventions, and previous studies have demonstrated the feasibility and potential efficacy of this approach [29,30]. In a systematic review of various mobile health (mHealth) interventions, Muessig et al [29] noted that internet- and mobile–based interventions can increase dissemination of HIV prevention interventions to wider populations while also providing consistency and lower cost in intervention delivery once fully developed. In addition, mHealth tools could promote behavior change and improve aspects of the HIV care continuum, including linkage to care, retention in care, and adherence to both PrEP and antiretrovirals [30,31]. As such, a mobile phone app that aims to increase HIV testing and PrEP uptake among YMSM has the potential to provide greater access to and uptake of these prevention services for this population.

### MyChoices App

**Figure 1:**
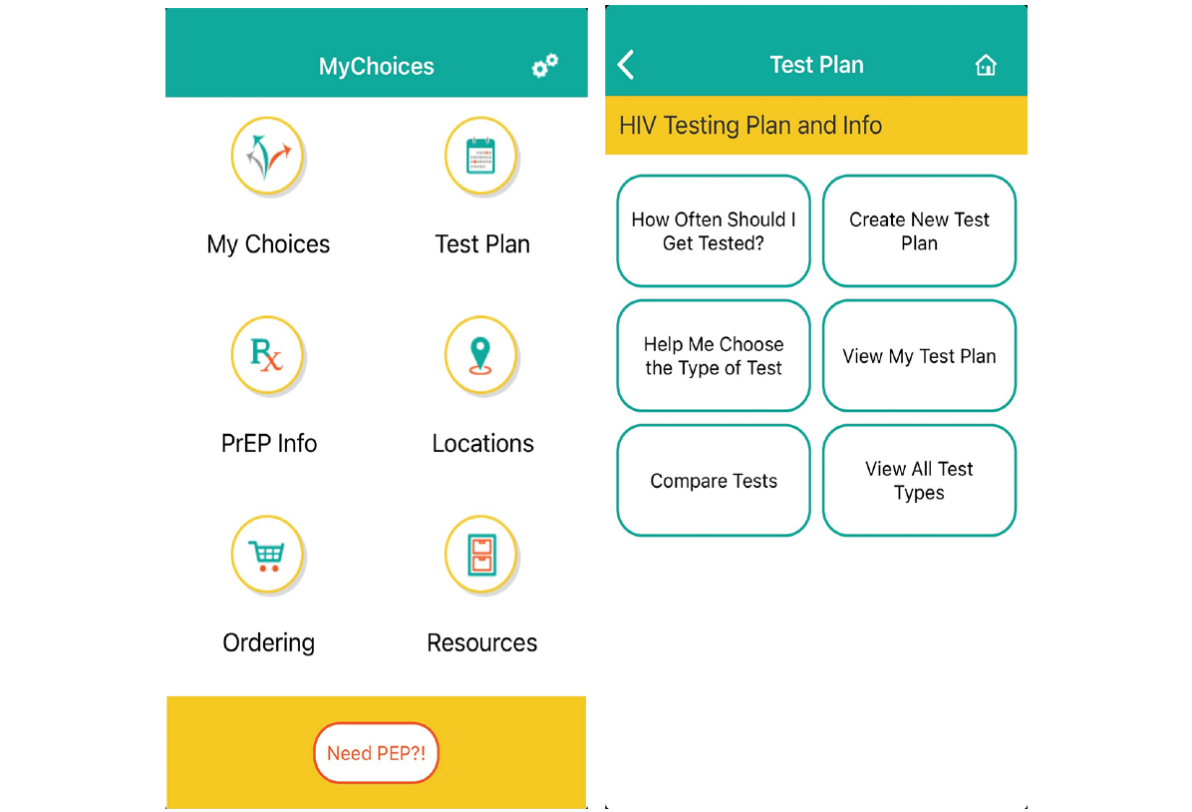
MyChoices app screenshots.

The development of the MyChoices app has been described previously [32]. In brief, MyChoices is a social cognitive theory–driven mobile app adapted from HealthMindr, an HIV prevention app developed through an iterative process for adult populations of MSM [33-35]. MyChoices built upon the initial framework and was subsequently adapted for youth by an interdisciplinary team of researchers with input from a diverse sample of YMSM at every stage of adaptation and refinement. The goal of the app is to increase HIV testing and PrEP uptake among YMSM in the United States by supporting goal setting, increasing self-efficacy, and enhancing self-regulation [36,37]. Key features of the MyChoices app include facilitation of the development of HIV testing plans with personalized recommendations; inclusion of reminder systems for HIV testing and GPS-enabled maps with local HIV and sexually transmitted infection (STI) testing locations and PrEP providers; ability to order free condoms, condom-compatible lubricants, and at-home HIV and STI test kits; and sexual health information using a variety of media (eg, videos, Graphics Interchange Format [GIF], infographics, frequently asked questions [FAQs], and quizzes) [32]. Figure 1 shows the MyChoices app home screen and the testing plan feature.

## Methods

### Study Population

Eligible participants were cisgender men who were aged 15-24 years; did not have an HIV test in the past 3 months; self-reported being HIV uninfected or HIV status unknown at screening; owned an iOS or Android mobile phone and were willing and able to download the MyChoices app; were able to understand, read, and speak English; were not taking PrEP; and had self-reported evidence of being at risk for HIV acquisition (details on risk criteria are given in the study by Biello et al [32]).

Through the University of North Carolina/Emory Center for Innovative Technology (iTech) [38], a part of the National Institutes of Health’s Adolescent Medicine Trials Network for HIV/AIDS Interventions [39], participants were recruited across 2 sites: Boston, Massachusetts (study site: Fenway Health), and the Bronx, New York City (study site: the Adolescent AIDS Program at Montefiore). Recruitment methods included posting on social media (eg, Craigslist, social networking ads, and gay networking mobile apps); distributing posters, flyers, and palm cards about the study; direct outreach at local venues frequented by YMSM (eg, community-based organizations, schools, bars, health fairs, and balls); and clinic-based recruitment.

### Theater Testing for App Refinement

After an initial prototype of the MyChoices app was developed through multiple rounds of formative research with YMSM [33,40], we conducted theater testing with 28 YMSM in 4 groups across the 2 iTech sites (5-8 participants per group). Theater testing allows for groups of participants to interact with the product being tested and provide feedback in situations that approximate real-life experiences and has been used commonly in mHealth app development [33,35,41,42]. Testing was conducted in a private room at each site by a research staff member who had training in qualitative methods and group facilitation. All participants completed a written informed consent or assent process before data collection commenced. Participants completed a brief demographic and behavioral questionnaire before theater testing to contextualize the group data collected. During theater testing, participants interacted with the MyChoices app prototype and provided feedback on the functionality, appearance, and usability of the platform. We also asked participants to comment on ways to maximize acceptability (eg, update language and improve flow) and to identify the components of the app that were liked or disliked and aspects that could be improved to increase HIV testing and PrEP uptake among YMSM. Groups lasted 60 minutes to 90 minutes and were audio recorded and professionally transcribed verbatim. Participants received US $50 as reimbursement for their time.

Members of the iTech Analytic Core [43] reviewed transcripts for quality and identified emergent themes. We then used Dedoose Version 8.0.35 (SocioCultural Research Consultants, LLC) software to apply the final codes to all transcripts. Thematic analysis involved using a primarily deductive approach to synthesize data coded for app acceptability, particularly around functionality, appearance, usability, and potential for improving HIV testing and PrEP uptake [44,45]. Findings are illustrated in the following sections using representative quotes. These data were used to refine the app before the initiation of the open technical pilot.

### Technical Pilot to Assess Feasibility and Acceptability of the MyChoices App

Once the MyChoices app had been refined, a technical pilot with 11 YMSM across the 2 iTech sites was conducted to assess the initial feasibility and acceptability and to identify any final areas for improvement. Eligible participants (mentioned earlier) attended a visit at the study site, at which they completed a web-based behavioral and psychosocial assessment, which included measures to assess sociodemographics (ie, enrollment city, age, race or ethnicity, educational status, and insurance status), sexual behaviors (ie, frequency of condomless anal sex), HIV testing history, and PrEP awareness. Study staff then assisted youth with app download and provided them with brief instructions on the purpose of the MyChoices app and an overview of how to use it; participants were encouraged to use the app over the course of 2 months.

At month 2, the participants completed a web-based assessment. In addition to the measures collected at baseline, we assessed *the acceptability* of the MyChoices app using the System Usability Scale (SUS) [46]. SUS is a validated 10-item measure that assesses the subjective usability of a system or, in this case, an app [46]. SUS has been extensively used in mHealth research and provides reliable results even with small sample sizes [47]. It is scored from 0 to 100, and a score of ≥50 indicates that the app is acceptable [48]. *Feasibility* was assessed using app analytics to determine whether the app was used, how often it was used, and what components were used most and least frequently.

Finally, we conducted *exit interviews* with participants to obtain feedback on app functionality, technical performance, errors and software bugs encountered, overall experiences using the app, feedback for further refinement, and subjective impact of the app on HIV testing and PrEP uptake. Exit interviews were conducted by study staff on the web using videoconferencing technology that was compliant with the Health Insurance Portability and Accountability Act. Interviews were transcribed and analyzed using the same approach outlined above.

Participants received US $50 for completing the baseline visit, US $25 for the 2-month assessment, and US $50 for the exit interview.

The study procedures were reviewed and approved by the University of North Carolina Institutional Review Board (IRB) as a single IRB-of-Record. IRB authorization agreements with all participating research entities were enacted. The MyChoices protocol is registered at ClinicalTrials.gov (NCT03179319).

## Results

### Findings From Theater Testing

Table 1 provides the characteristics of the 28 YMSM who participated in theater testing. Participants’ ages ranged from 16 years to 24 years, with a mean age of 20 years. YMSM of color made up 57% (16/28) of the sample, and 57% (16/28) of the participants were still in school.

A total of four key themes emerged from theater testing, and thus, we structure the presentation of our results to highlight these findings: (1) general utility and acceptability of the app, (2) feedback and suggestions for user interface, (3) opinions on language, and (4) suggestions for additional content and features.

**Table 1:** Demographic characteristics of participants in theater testing of a novel HIV prevention app for HIV prevention in young men who have sex with men in Boston, Massachusetts, and the Bronx, New York City, 2019 (N=28).

Characteristics		Values
Age (years), mean (SD)		20 (2.0)
Number of condomless anal sex acts in the past 6 months, mean (SD)		10 (15.4)
**Study site, n (%)**		
	Boston, Massachusetts	15 (54)
	The Bronx, New York City	13 (46)
Hispanic or Latinx, n (%)		7 (25)
**Race, n (%)**		
	Black	10 (36)
	White	12 (43)
	Multiracial or other	6 (21)
Currently in school, n (%)		16 (57)
**Highest level of education completed, n (%)**		
	Less than high school	4 (14)
	High school diploma or graduate equivalency degree	8 (29)
	Some college, or technical or vocational school	13 (46)
	Four-year college graduate or more	3 (11)
Currently has health insurance, n (%)		24 (86)
HIV test in the past 3 months, n (%)		19 (68)
Heard of PrEP^a^ before the study, n (%)		26 (93)
**Ever used internet or apps for the following (not mutually exclusive), n (%)**		
	Tracking health behaviors	15 (54)
	Getting information about HIV or other STDs^b^	13 (46)
	Getting other health or medical information	15 (54)
	Sending reminders	21 (75)

^a^PrEP: pre-exposure prophylaxis.

^b^STD: sexually transmitted disease.

#### General Utility and Acceptability of MyChoices

Participants described that an HIV prevention app needs to be perceived as useful to its target audience and that they saw value in MyChoices. The participants described:

Yeah, I feel like perceived value...in terms of repeated use, you sort of have to make sure that people get into using it for like reminders or like plans. Otherwise, there’s no reason to go back. Otherwise it’s just like an information center that you could find on the Internet, you know?

White, gay, age 21 years, Boston

I think like this app is very, very helpful and useful. Like I like how the questions are not too personal. Like they’re the same questions that doctors would ask you. And I like how you can take a survey and it shows when you should get tested, like every three months or something like that. And the ordering, I think that is very smart. And the location, that is very smart too. And I just like – I like this app overall.

Black, gay, age 16 years, the Bronx

Although participants acknowledged that they could find similar information from other sources, they appreciated that the information on MyChoices came from a reliable source because of its association with the study and clinic and that using the app was a better avenue for ascertaining health information than searching the internet for resources they could not be sure were accurate:

I think the idea of this app is great. Giving MSM, you know, PrEP information and PEP [post-exposure prophylaxis] information and easier ways to access different locations and information is great, you know, because a lot of us aren’t as educated on these topics and aren’t—don’t have these resources. And using the Internet, it’s way harder to access the information just by looking it up on Bing or Google than, you know, the app just gives it to you, you know?...It definitely gives you a lot of information that you wouldn’t have access to otherwise.

Afro-Latinx, bisexual, age 20 years, the Bronx

Participants identified a number of features that they believed would be most useful, including the ability to order free HIV or STI test kits, condoms, and lubricant; being able to create personalized HIV testing plans with the location finder; and being able to ask questions to a health professional through the app:

I would say that this is the most immediately pertinent thing for me that I’ve seen in the app so far. I think that it’s really important to have a plan when it comes to getting tested and to know, like have a schedule and things like that.

Specific identity unknown

The map features are working well here...when it’s full screen I really like it. I love using maps for everything in my life. And I love seeing like where I am and where I could go.

White, queer, age 22 years, Boston

#### Suggestions for User Interface

Many participants liked the *look and feel* of the app, noting that it was nondescript enough to sufficiently maintain privacy:

You don’t want it [the app] to be something that someone just scrolling through your homepages will be like, “Oh, that’s what that is.” [an HIV prevention app] It [the app] doesn’t really show that particularly.

White, gay, age 24 years, Boston

Participants appreciated the wide range of media types, including colorful icons, GIFs, videos, and text. One participant described a GIF related to accessing PrEP:

I feel like they're great animations to, you know—it simplifies what it actually is. It’s showing you what you're doing. Like, the individual, it shows you coming from your house, going to the doctor’s office and then, you know, talking to the doctor.

Afro-Latinx/Asian, age 23 years, the Bronx

However, others expressed that the colors and format made the app appear *basic*:

I think it looks sort of basic and plain. Particularly maybe just because of the white background...It makes it look a bit sort of less mature like this and less formal. Which is perhaps not the vibe I’d look for in a health app.

Asian, gay, age 21 years, Boston

Some participants suggested that portions of the app felt repetitive and could be better streamlined and organized:

I think there are too many icons. Because I tapped the “my activity” button and that led me to ordering condoms. So I think if the choices were more simplified, I think I would understand a little bit more about what I can do with the app.

White, gay, age 21 years, Boston

Conversely, others felt that obtaining similar information in a variety of different ways was helpful and a strength of the MyChoices app:

I think that it’s good to kind of have like a no wrong door approach to it where you can get to it in a variety of ways.

White, gay, age 20 years, Boston

#### Feedback on Language

Some participants felt that the language used was appropriate and made them trust the source:

And, yeah, I just think overall like it was actually really medically-driven too which I like that. It kept it very professional.

Latinx, pansexual, age 18 years, the Bronx

However, others felt that the language used to present the information was too academic and included too much science jargon:

Just, I have a part about comfortability. I think I want to feel comfortable when I'm using it...This is just something friendly, it’s supposed to be a guide or resource. It’s not supposed to scare me or freak me out.

Black, queer, age 19 years, the Bronx

Participants appreciated the places in the app that allowed them to personalize the language so that they could decide what type of language—more or less direct, more or less casual—suited them personally. They also felt that personalization of the language also protects against privacy and confidentiality concerns because you “can put whatever you want”:

But the on screen notification is cool. I mean, it’s gonna be more options. It depends. Everybody’s different, so everybody has a different preference to what they, how they like to be notified.

Afro-Latinx/Asian, age 23 years, the Bronx

#### Suggestions for Content and Features

In addition to feedback on the current MyChoices prototype, participants provided suggestions on additional content and functions that might enhance the acceptability and utility of the app. One participant suggested including a section on how to talk to your partner about PrEP:

I think that another resource that would be helpful on this page would also be talking to your partners about PrEP...If you’re having sex with multiple partners regularly then it might something to be like how to tell your partner that you are on PrEP. Or how to possibly suggest to a partner that going on PrEP might be a good option.

White, gay, age 24 years, Boston

Participants also felt that getting tested for HIV, and even talking about HIV, can produce a lot of anxiety and that the app could include more information about what to do if you do get a reactive test to assuage some of those fears:

...there should be something in there, like a section that should say, “Oh, if you do have it [HIV],” resources about that, about going to get help, like, there would be something that could help you get that.

Afro-Latinx, gay, age 20 years, the Bronx

Similarly, participants described their belief that many young people were still misinformed and lacked adequate knowledge about HIV. As such, they suggested including more basic information about HIV in the app:

...If we want to make this really accessible and make sexual health like an accessible topic, you sort of need to go back to basics, and be like, “Here is what HIV is, here’s what happens, here’s what it is not, here’s how to treat it.” And that sort of basic information, I think would really helpful to keep people going back to, “This taught me a lot,” you know.

White, gay, age 19 years, Boston

In addition to new content, participants included suggestions for new features. For example, we described a potential feature that would use geofencing to notify a user when they are near an HIV testing location and they are *due* for an HIV test according to their created test plan. Participants were excited about this option, saying “that’s pretty cool” and “that’s a cool feature.”

Participants also suggested additional tools for interacting with health professionals, including allowing HIV testing sites to provide results directly through the app:

Part of me wished that if – so let’s say, you got tested from [Health Center] and there was a way [Health Center] could coordinate so that your results just like pop-up in the app, like you don’t have to put it manually and that is like your way of receiving them, too. So it is all in one place and you don’t do it manually...And they just like get a push notification like, “Your results are in!” And that’s like – you go in and you just like find out that way.

White, gay, age 21 years, Boston

Participants also appreciated information about postexposure prophylaxis (PEP) but felt that the need for immediate action warrants easier access to this information:

It would’ve been great if like within this app there is like an, “I’ve just had unprotected sex. What do I do?” You know, because I think for it to come up only in the part about PEP is like, you know – or for it to be like the check-in and then, oh, in the past 72 hours, what if it has been like more than that 90 hours or something, because I didn’t like really, you know, have that time to like be checking a quiz.

Black, gay, age 20 years, Boston

#### Summary of Changes Made to MyChoices After Theater Testing

As noted earlier, the participants saw the value in the content and functionalities of the MyChoices app. They believed that the suggestions that came from brief quizzes were useful, that the testing plan would be helpful to encourage regular testing, that the PrEP information was instructive, and that being able to order HIV or STI test kits and safer sex supplies would be beneficial. However, participants also provided suggestions on how to build on and improve some of these components. As a result of these suggestions, before initiating the technical pilot, we refined some of the language in the app, updated GIFs and icons, and streamlined the flow through the app. Moreover, we expanded app functionalities to include (1) a geolocator function that pings individuals when they are near a testing site and due for HIV testing based on their personalized testing plan, (2) the *Need PEP?!* button that is available at the bottom of every screen on the app to directly connect participants with information about PEP and locations where it is available, (3) additional videos to demonstrate how to use the home testing kits, and (4) emails that are sent to users after downloading the app to introduce them to key features that they may have otherwise missed. Some suggestions made by participants were unable to be implemented into the app, although they were noted as potential ways to enhance future iterations, including receiving test results from clinics through the app, adding a real-time chat feature, syncing reminders with phone calendars, and being able to schedule HIV testing or PrEP appointments through the app.

### Findings From the Technical Pilot

The open pilot enrolled 11 participants (Boston, n=6; the Bronx, n=5), and retention at the 2-month follow-up was 100%. Participants’ ages ranged from 15 years to 23 years, with a median age of 19 years. YMSM of color made up 91% (10/11) of the sample (Black, non-Hispanic, n=5; Hispanic or Latinx, n=5; Table 2).

**Table 2:** Baseline demographic characteristics of participants in the technical pilot of a novel HIV prevention app for HIV prevention in young men who have sex with men in in Boston, Massachusetts, and the Bronx, New York City, 2019 (N=11).

Characteristics		Values
Age (years), mean (SD)		18.8 (2.7)
Number of condomless anal sex acts in the past 6 months, mean (SD)		4.0 (6.8)
**Study site, n (%)**		
	Boston, Massachusetts	6 (55)
	The Bronx, New York City	5 (45)
Hispanic or Latinx, n (%)		4 (36)
**Race, n (%)**		
	Black	7 (64)
	White	3 (27)
	Multiracial or other	1 (9)
**Sexual orientation, n (%)**		
	Gay or homosexual	7 (64)
	Bisexual	4 (36)
	Same gender loving	2 (18)
	Queer	1 (9)
Currently in school, n (%)		8 (73)
**Highest level of education completed, n (%)**		
	Less than high school	3 (27)
	Some college, or technical or vocational school	6 (55)
	4-year college graduate or more	2 (18)
Currently has health insurance, n (%)		11 (100)
Currently has primary care provider, n (%)		9 (82)
**HIV test, n (%)**		
	In the past 3 months	0 (0)
	Ever	3 (27)
**STI^a^** **test, n (%)**		
	In the past 3 months	0 (0)
	Ever	6 (55)
Heard of PrEP^b^ before the study, n (%)		9 (82)
Discussed PrEP with a health care provider before the study, n (%)		2 (18)
**Interested in taking PrEP, n (%)**		
	Somewhat or very or extremely interested	8 (73)
	A little interested	3 (27)

^a^STI: sexually transmitted infection.

^b^PrEP: pre-exposure prophylaxis.

#### App Feasibility

Participants in the technical pilot accessed the app, on average, 8 times (SD 5.0; range 2-18) over 2 months, with an average duration of 28 minutes per session (SD 38.9). Across all participants, the cumulative time spent in the app ranged from 1.8 minutes to 20.5 hours, with an average of 4 hours and 39 minutes (SD 7 hours). All participants used the test plan feature (11/11, 100%; average number of accesses to this feature 20.2, SD 18.2; range 1-63), and nearly all participants (10/11, 91%) used MyChoices to order HIV or STI self-testing kits and safer sex supplies (average number of accesses 22.6, SD 29.8; range 0-85). Most participants (7/11, 64%) used the app to locate HIV or STI testing centers or PrEP providers (average number of accesses 5.3, SD 5.8; range 0-17), and 4 participants (4/11, 36%) accessed the FAQ feature of the app to access information about HIV prevention and PrEP (average number of accesses 1.5, SD 3.0; range 0-9).

#### App Acceptability

At the 2-month follow-up, the mean SUS (0-100) score was 71 (SD 11.8), which is considered above average. Almost all participants (9/11, 82%) agreed that MyChoices was useful, 73% (8/11) were very satisfied with MyChoices, and 91% (10/11) said that they would recommend it to a friend who needed help with getting HIV tests or accessing PrEP. Nearly all participants (9/11, 82%) reported that they would be very likely (2/11, 18%), likely (4/11, 36%), or somewhat likely (3/11, 27%) to use the MyChoices app if it were to become publicly available. The utility of MyChoices for HIV testing and PrEP was also highly rated (Table 3).

**Table 3:** Utility of the MyChoices app for HIV prevention in young men who have sex with men in Boston, Massachusetts, and the Bronx, New York City, 2019 (N=11).

Dimension	Values^a^, n (%)
MyChoices motivated me to get tested for HIV	10 (91)
MyChoices helped me understand whether PrEP^b^ would be a good fit for me	9 (82)
MyChoices assisted me in getting tested for HIV	8 (73)
MyChoices helped me understand my risk for getting HIV	7 (64)
MyChoices assisted me in getting started on PrEP	5 (45)
MyChoices motivated me to get on PrEP	5 (45)

^a^Number (percentage) of participants in the technical pilot who indicated that they strongly agreed or agreed with each of the statements.

^b^PrEP: pre-exposure prophylaxis.

The most highly rated features of the MyChoices app in terms of helpfulness were the ability to order self-testing kits and safer sex supplies (10/11, 91%), PrEP information (10/11, 91%), HIV or STI and PrEP locator (9/11, 82%), and personalized testing plans (8/11, 73%). Less helpful features were the check-in quizzes (6/11, 55%) and testing reminders (5/11, 45%). Finally, participants agreed that the MyChoices app had a beneficial impact on their lives in the following ways: getting tested and knowing their HIV status (8/11, 73%) improved their understanding of the risk of HIV (7/11, 64%), feeling good about helping others or community (6/11, 55%), getting access to medical care (6/11, 55%), receiving assistance for getting on PrEP (5/11, 45%), and improving personal relationships (4/11, 36%).

#### Exit Interviews

All 11 participants completed follow-up exit interviews. Overall, there was a high level of acceptability of the content, interface, and features. Participants commented on the relevant information presented in the app, one participant noting that “[they] learned a lot about PEP and PrEP” and noted that the app facilitated their scheduling a PrEP counseling appointment with their primary care provider. Another participant noted that they “didn’t know anything about [PrEP] until [they] used the app.” Furthermore, the feature to schedule PrEP appointments “was the best part for [them]” because “it shows you, like, you are and, like, the different centers, testing centers around you, the different communities around you.” The participant endorsed how the app helped him feel more connected to the community where they lived. Another participant highlighted how the app provided him with helpful information about health clinics in his area, of which he was unaware despite living in the neighborhood “a good portion of [his] life.”

Participants also provided suggestions to improve the app and provided specific feedback on additional resources and features that they found interesting and potentially helpful. One participant suggested including a *myth busting* section in the app to offset false information spread on the web about sexual health. A few participants also noted that some of the information in the app remains repetitive. One participant suggested defining PrEP differently in different areas of the app to reduce repetition and to mitigate a potential lack of understanding of health information.

## Discussion

### Principal Findings

This study describes the findings from two phases of formative data collection (theater testing of the MyChoices mobile app prototype and the technical pilot of MyChoices fully functional app) to create a mobile app to increase HIV testing and PrEP uptake among young MSM in the United States. HIV prevention apps are proliferating; however, using evidence-based methods for intervention development, including community-centered approaches with iterative feedback from the community, is essential to maximize their impact and reach [35,49], and few HIV prevention apps for youth have been developed and tested using the approaches described in this paper.

In the context of previously reported findings about preferences for mobile HIV prevention apps in young people, our findings support and extend previous reports by providing a sample including a higher proportion of teenage participants, confirming some aspects of previous reports, and documenting some novel findings. Like others [50-52], we found that youth valued having an app that presents credible information relevant to their health and interests in direct and understandable language and the value of having educational information available. Notably, other qualitative studies of preferences for HIV prevention or HIV management eHealth tools have identified some themes that did not emerge from the youth who participated in our study. For example, other researchers have found youth preferences for features that allow them to interact with other youth [53], and young MSM in other studies reported concerns about privacy and confidentiality of data [54]. Young MSM have also raised questions about what the scope of sexual health apps should be, with some suggesting that related health issues, such as substance use, could be included in a mobile app resource [52]. Our participants also provided suggestions for developing geospatial tools to guide users to prevention services. These issues, which have been rarely reported in other studies, may have emerged from our participants because geospatial tools are becoming more refined and younger people have grown up relying on smartphones as a primary source of navigation. Due to the younger age of our participants compared with many previous studies and because our data were collected more recently, youth expectations around enhanced navigation and geolocation services may be seen as emerging expectations for mobile prevention apps.

Theater testing revealed high levels of interest in the content provided through MyChoices, with youth indicating that the availability of this type of information is both lacking and necessary. Although youth have access to huge amounts of health information through the internet and report using the internet frequently to access this type of information [55,56], they do not always know what to trust [57,58]. In theater testing, youth indicated that one of the major strengths of the app was to have access to a wide range of sexual health information in one place, in multiple formats, that they knew they could trust. In addition, the app’s usability score is comparable with the HealthMindr app on which MyChoices was based, although it had a higher proportion of people who would recommend it to friends [33]. This may suggest a successful adaptation for YMSM.

In addition to the content, the features of the MyChoices app were also broadly seen as favorable and useful. In both theater testing and the technical pilot, the most popular features included ordering free HIV and STI self-testing kits, condoms, and lube; ability to search for nearby HIV testing and PrEP care sites; and PrEP information provided in multiple formats (ie, text, videos, GIFs, and infographics). This suggests that YMSM are open to accessing multiple means of HIV prevention support and that providing a large toolbox of HIV prevention options using diverse modalities is essential for reaching this group at the highest risk for HIV acquisition [59].

Although there was consensus on the importance of the content and utility of the features of MyChoices, there was a wide range of views on preferences for the user interface. Some participants appreciated that the language and presentation of information was more *formal*, as it was viewed as more trustworthy and legitimate. However, others felt that the interface was bland and potentially even anxiety producing and that more casual language would render the app more relatable. This dichotomy highlights the ongoing difficulty in developing an app that aims to reach large populations; although YMSM in the United States are a subpopulation of a larger group, they are not a monolith and will have diverse needs and preferences [60-62]. In addition, with technology constantly changing and advancing, mobile apps must be flexible and responsive to these changes in technology and end user preferences [63,64].

### Limitations

These results should be interpreted in light of the following limitations. First, the technical pilot was small and used a nonrandomized design, and as a result, it was not powered or designed to evaluate efficacy. Sample sizes for technical pilots are often determined based on practical considerations rather than inferential statistical power calculations [65]. Still, the evaluation of feasibility and acceptability of behavioral interventions in open pilot studies is an important part of the intervention development process and helps inform subsequent randomized controlled trials (RCTs) to evaluate the efficacy of the intervention [66]. Second, social desirability bias may have led participants in the theater testing and technical pilot to speak more positively about their experience of the app during focus groups and exit interviews. To minimize the potential for these biases, participants were continuously reminded that there were no right or wrong answers and that it was important to provide honest responses. Third, for both phases of the study, individuals were enrolled in the Bronx, New York City, and Boston, Massachusetts, only, potentially limiting the generalizability of the findings. Future studies should expand to other regions of the country and outside of large metropolitan areas, particularly in the South, where the HIV epidemic is spreading most rapidly among young Black MSM. Finally, in both phases of the study, participants had to report sexual risk for HIV, no current PrEP use, and no recent HIV testing. This allowed us to ensure that we received input from those at the highest risk and from those who may benefit the most from the app; however, it also limits the generalizability of our findings to less risky populations.

### Conclusions

HIV incidence in the United States remains disproportionately high among YMSM, compared with other risk and demographic groups. YMSM are also less likely than their adult peers to know that they are infected with HIV, highlighting an imminent need to increase routine HIV testing and expand access to HIV prevention interventions, including PrEP. Smartphone use is ubiquitous in the United States, and mobile apps offer an opportunity to reach YMSM “where they’re at.” mHealth apps have proliferated in recent years; however, only a limited number have been theory-driven and developed using evidence-based methods for intervention development. These data from theater testing and a technical pilot show the initial promise, feasibility, and acceptability of the MyChoices app to improve HIV testing and PrEP uptake among YMSM in the United States. The next step involves further pilot testing using an RCT design to determine more accurate effect size estimates; a full-scale RCT efficacy trial; and ultimately, if efficacious, an implementation study to ensure it is disseminated in such a way that maximizes its reach and utility. At each step, iterative evaluation and refinement based on the reflections and experiences of YMSM will be prioritized.

## Abbreviations

FAQ: frequently asked question
GIF: Graphics Interchange Format
IRB: Institutional Review Board
iTech: University of North Carolina/Emory Center for Innovative Technology
mHealth: mobile health
MSM: men who have sex with men
PEP: postexposure prophylaxis
PI: principal investigators
PrEP: pre-exposure prophylaxis
RCT: randomized controlled trial
STI: sexually transmitted infection
SUS: System Usability Scale
YMSM: young men who have sex with men
